# Menstruation and the Cycle of Poverty: A Cluster Quasi-Randomised Control Trial of Sanitary Pad and Puberty Education Provision in Uganda

**DOI:** 10.1371/journal.pone.0166122

**Published:** 2016-12-21

**Authors:** Paul Montgomery, Julie Hennegan, Catherine Dolan, Maryalice Wu, Laurel Steinfield, Linda Scott

**Affiliations:** 1 Centre for Evidence-Based Intervention, University of Oxford, Oxford, United Kingdom; 2 SOAS, University of London, London, United Kingdom; 3 Applied Technologies for Learning in the Arts and Sciences, University of Illinois, Urbana-Champaign, Illinois, United States of America; 4 Bentley University, Waltham, MA, United States of America; 5 Said Business School, University of Oxford, Oxford United Kingdom; Iranian Institute for Health Sciences Research, ISLAMIC REPUBLIC OF IRAN

## Abstract

**Background:**

Poor menstrual knowledge and access to sanitary products have been proposed as barriers to menstrual health and school attendance. In response, interventions targeting these needs have seen increasing implementation in public and private sectors. However, there has been limited assessment of their effectiveness.

**Objectives:**

Assess the impact of providing reusable sanitary pads and puberty education on girls’ school attendance and psychosocial wellbeing outcomes.

**Methods:**

A cluster quasi-randomised controlled trial was conducted across 8 schools, including 1124 girls, in rural Uganda. Schools were allocated to one of four conditions: the provision of puberty education alone; reusable sanitary pads alone; puberty education and reusable sanitary pads; and a control (no intervention). The primary outcome was school attendance. Secondary outcomes reflected psychosocial wellbeing.

**Results:**

At follow-up, school attendance had worsened for girls across all conditions. Per-protocol analysis revealed that this decline was significantly greater for those in the control condition *d* = 0.52 (95%CI 0.26–0.77), with those in control schools having a 17.1% (95%CI: 8.7–25.5) greater drop in attendance than those in any intervention school. There were no differences between the intervention conditions. High rates of school drop-out and transfer meant the trial suffered from substantial participant drop-out. Intention-to-treat analyses using two different imputation strategies were consistent with the main results, with mean differences of 5.2% attendance in best-case and 24.5% in worst-case imputations. Results were robust to adjustments for clustering. There was no impact of the interventions on girls’ self-reported shame or insecurity during menstruation.

**Conclusion:**

Results of the trial support the hypothesised positive impact of providing sanitary pads or puberty education for girls’ school attendance in a developing country context. Findings must be interpreted with caution in light of poor participant retention, intervention fidelity, and the attendance measures used.

**Trial Registration:**

Pan African Clinical Trials Registry PACTR201503001044408

## Background

Educating girls has been proposed as the world’s highest yielding investment for developing countries. [1] Improved educational attainment has been consistently linked to economic growth and productivity.[2, 3] For women and girls, increased education has been associated with benefits including: health for women and their children, literacy, delayed sexual debut and marriage, self-efficacy, improvements in labour force participation, and involvement with household decision making.[1, 4–8] These benefits rise substantially with increasing years of schooling.[1]

Global improvements in gender parity in school enrolment have been considerable. UNESCO [9] has reported that at the end of 2015, 69% of countries with data had achieved or were likely to reach gender parity in primary education. Disparities are greater in secondary education where less than 50% of countries are projected to reach gender parity.[9] Poverty deepens disparities, with poor girls the least likely to enter or complete primary and secondary schooling.[9] In Uganda, the context of the present study, high levels of enrolment in primary school drop off sharply in secondary school.[10–12] In 2013, figures suggested only 22% of school-aged girls were enrolled in secondary school in comparison to a 91% enrolment rate in primary school.[9, 12, 13] Gender parity in school enrolment differed by area, with disparity increasing with rurality.[12] While enrolment figures represent easily available and comparable statistics, it is the quality of education as well as attendance and engagement at school which contribute to girls’ ability to learn effectively when enrolled, and thus garner the benefits of education.[1, 9, 14]

Many barriers to girls’ school attendance, participation, and retention have been identified.[6, 15] Cultural expectations, household responsibilities, early pregnancy and marriage, and prioritisation of boys’ education have all been discussed.[9, 16] Further, school environment barriers to education for girls include a lack of female teachers, sanitation and hygiene facilities, and gender-based violence.[6, 17] Menstruation has emerged as an additional gender-specific barrier to school participation amongst adolescent girls.[18] Particularly for poorer girls, the most disadvantaged in access to education, the management of menses presents a significant obstacle to health, comfort, and engagement with school.

Qualitative studies have revealed the construction of menstruation as embarrassing, shameful, and dirty across many contexts.[19–21] Taboos around the topic mean many adolescent girls are unprepared for menarche, and that management practices are not discussed openly.[19, 20, 22] In the confined school environment, with a lack of access to adequate latrines or separate latrines for males and females, menstrual management presents a significant challenge. Absorbents such as cloth are used and can often leak, soiling uniforms or outer garments. In addition, cleaning and changing absorbents pose significant challenges. Girls interviewed have described feelings of shame, fear and distraction associated with menstruation. Both girls and teachers have expressed the view that menstruation is a barrier to school attendance and engagement.[19, 21]

Quantitative work has begun to support these assertions, although estimates have varied. A study of 595 school girls in northeast Ethiopia found that after adjustment for residence, household income and parental education, girls who did not use disposable sanitary pads had 5.37 times higher odds of school absenteeism during menses than those using disposable pads.[23] In contrast, a survey study in rural Malawi of 717 girls aged 14–16 reported that after adjusting for sociodemographic characteristics and other reasons for school absenteeism, menstruation did not predict absence.[24] Intervention studies have also provided mixed support for the association between menstruation and absenteeism.[25–27]

Studies have hypothesised that both physical barriers and a lack of menstrual knowledge to support hygienic management of menses contribute to disengagement and absenteeism.[28–30] Thus the provision of clean menstrual absorbents, and information about the effective and hygienic management of menstruation, have been proposed as simple and efficient interventions to improve the comfort, dignity, and school participation of adolescent girls. Increasingly governments, charities, and development organisations have sought to target menstruation as a barrier to school attendance.[31–33] However, there is sparse evidence for the effectiveness of such interventions.[34] The first randomised controlled trial, conducted in Nepal, used girls’ menstrual calendars (self-kept) and attendance records to estimate that only 0.4 days in 180 were missed due to menstruation.[25] The study found no improvement in attendance after providing menstrual cups. Conversely, two pilot non-randomised trials in Ghana and Kenya respectively found interventions providing sanitary pads and education, or materials and education for girls to make their own pads improved school attendance.[26, 27] There is a need for larger, randomised trials to provide causal evidence for the link between menstruation and education, as well as the effectiveness of proposed interventions.[34]

In 2009 this research group undertook a pilot trial of sanitary pad provision and puberty education in peri-urban and rural Ghana. The non-randomised cluster-control trial of 120 schoolgirls (aged 12–18) across four schools tested the effectiveness of the provision of disposable sanitary pads and/or a puberty education class on school attendance over a 5-month period. Results were promising with a 9% increase in attendance found for those receiving pads and education (see [26]). Qualitative work indicated improvements in shame and self-confidence.[22] Whilst findings of the pilot were positive, they were limited by a lack of randomisation, small sample size, comparison of single clusters, and short study duration. In addition, the trial raised questions with regard to the effectiveness of providing sanitary pads in contrast (or addition to) education, and the need to identify the individual impact of these interventions.[26]

The present study represents a scale-up from the initial pilot to a cluster quasi-randomised control trial of reusable sanitary pad and puberty education provision. A Latin-square design meant four different conditions were compared to isolate the individual and combined effect of pad provision and puberty education. Clusters received one of the following interventions: reusable pads alone, education alone, reusable pads and education, or a no-intervention control.

## Objectives

This trial aimed to assess the impacts of providing reusable sanitary pads and puberty education on school attendance and psychosocial outcomes for girls in Uganda. In addition, the study seeks to isolate the impact of resource provision (pads) and education as potential interventions to reduce menstrual-related absence and consequences for psychosocial wellbeing.

## Methods

The trial was registered with the Pan African Trials Registry (PACTR201503001044408) (S1 File). Administrative challenges meant the trial was not registered until after follow-up. The trial was registered prior to the analyst receiving any data, and details were recorded consistent with the protocol prior to the start of the trial to ensure transparency around deviations, noted below. The authors confirm that all ongoing and related trials for this intervention are registered. Outcomes reported in the paper are the same as those registered in the protocol. The trial is reported in accordance with CONSORT (cluster RCT) guidance [35] (for checklist see S1 Table).

### Context

The intervention took place in Kamuli district, located in the eastern-central region of Uganda, where the partner NGO (Plan International) operates several health and education programmes. The district was selected due to its poor performance across a range of key education, health and welfare indicators, with the district characterised by “high school dropouts, high illiteracy rates, high levels of morbidity and mortality, early marriages, high fertility rates, intermittent food shortages and malnutrition, and poor housing”.[36] The district’s literacy rate continues to lag behind the national average (61.8% vs 70%, respectively), with significant disparities between the literacy of girls (54.6%) and boys (69.7%).[36] Poor literacy rates are particularly pronounced among children; the District has the 3^rd^ lowest rates of primacy school literacy in the country (14% compared to 28% nationally).[37] Approximately a third of students never complete primary school, and the majority of those who do, do not progress to secondary school.[38]

### Design

The trial was conducted between January 2012 and December 2014. The initial sample size included in the protocol (n = 560) was calculated based on the effect size (partial-eta squared = 0.094) and drop-out (15%) from the Ghana pilot [26] to achieve 80% power at a 5% α-level, but clustering was not accounted for. Eight rural primary schools were recruited into the study through the partner NGO. Budget limitations precluded the enrolment of additional schools. Schools were purposively selected from the Kamuli district as described above. Schools were required to be within reasonable distance from the partner NGO, and to be comparable in major characteristics such as degree of rural location and distance from services, size, educational provision, toilet facilities, and education quality based on the extensive local knowledge of the partner NGO. Additional summary of school facilities is provided in S2 Table. School clusters were selected to have sufficient geographical separation so as to reduce risks of contamination. In each school, girls in primary grades 3 to 5 (ages approx. 10–13, but extending above this) were enrolled in the study. Girls were not excluded based on menstrual status as the trial aimed to capture both menstruating girls and those who were likely to commence menstruating during the study (a very early protocol deviation). Written consent for participation was sought from girls and a parent/caregiver; none declined to participate. A total of 1124 girls were recruited into the trial prior to baseline.

The study was a four-armed cluster quasi-randomised control trial. After baseline attendance data collection and the baseline survey had been completed (see below), schools were grouped into pairs maximising the distance between the clusters. The four clusters were then quasi-randomised to conditions (two schools per condition). Clustered schools were allocated sequentially in alphabetical order of the first school in the pair to one of the four conditions:

1. The provision of puberty education
2. The provision of reusable sanitary pads (AFRIpads)
3. The provision of puberty education and reusable sanitary pad (AFRIpads)
4. A control condition (receiving no pads or education).

After allocation, neither the intervention delivery team nor participants were blind to intervention condition. The clustered design prevented contamination of education or pad distribution, and prevented social and ethical issues which may have arisen by unequally distributing resources to some girls and not others in a single community. Cluster sizes varied depending on the number of pupils in each school, as all girls in primary grades 3–5 were included.

### Intervention

#### Development

The trial followed a pilot in Ghana, however, many modifications were made. In particular, the change from commercial disposable sanitary pads, to a locally-made, reusable AFRIpad. Among the key limitations of the pilot work were the environmental consequences of pad disposal.[22, 26] Further, the cost of monthly provision of disposable pads was substantial. A reusable product was believed to be more sustainable, both from a cost and environmental perspective. Prior to selecting AFRIpads, an acceptability study was conducted comparing three different types of reusable pads, the results of which have been reported elsewhere.[39] Girls in the study reported that the reusable pads were more reliable and stable than their previous methods of menstrual absorption (e.g., cloth, underwear alone), and AFRIpads were thus selected as the product of choice for the trial.

#### AFRIpads

Girls in the conditions receiving pads were provided with a pack of AFRIpads, 3 pairs of underwear, and a small quantity (one sachet, 45grams) of Omo (soap) with which to wash the pads. Pads were distributed in October 2012, and again in March 2014. When distributing the pads, girls were also taught about their correct use and cleaning by locally trained NGO research assistants. AFRIpads are a washable, reusable cloth pad produced in Uganda. They are manufactured by a social business in Uganda that specializes in locally-produced, eco-friendly sanitary pads. Pads are made of quick-drying fleece and sold in a kit that includes two soil-resistant plastic-lined ‘base’ pads that fasten securely to underwear, three attachable winged liners, three straight liners and two small bags for carrying. AFRIpads can be reused for 12 months ([39]; http://www.afripads.com/).

#### Puberty education

Locally trained community health nurses from the partner NGO administered the education intervention in October 2012. Education sessions were conducted in each school and lasted approximately 1.25hrs. Education followed the Straight Talk training guide developed by the Straight Talk Foundation Uganda (http://straighttalkfoundation.org/). Education covered puberty changes, menstruation, early pregnancy, life skills, prevention of HIV, strategies for avoiding sexual assault, healthy relationships, and friendship formation and goal setting. This education programme was selected for its high face-validity concerning the topics of puberty in the study. It has a small but promising evidence base, and available community health nurses had familiarity with the program. It was deemed contextually appropriate and endorsed by project stakeholder groups. The manual from the program can be downloaded from the intervention program page (http://www.spi.ox.ac.uk/fileadmin/documents/PDF/Straight_Talk_Plan.pdf).

### Intervention delivery and protocol deviations

The sample size recruited was larger than reported in the original protocol. Based on the results of the pilot study, power analysis calculated the total sample size requirement to be 560 girls across the eight schools. However, attendance was taken for all girls in the assigned classes (primary grades 3 to 5). As there was a much higher rate of drop-out over the 2-year study period than in the pilot (only 5 months), this additional data collection meant sufficient numbers were still available at the 24-month follow-up to detect an effect. Far fewer girls were reached for survey at the final time-point than intended, despite the administration of a drop-out survey and attempt to follow up girls who were no longer in the study schools. Resource restrictions limited the amount of time that could be dedicated to finding girls who had dropped out of the study schools, thus assessments of those who did and did not drop-out are limited to baseline measures. There was a very early change to the protocol to include menstruating and non-menstruating girls, as noted above, in an attempt to reach girls before their first menses.

Significant errors in intervention delivery occurred across the trial. The intended protocol was for all girls in education conditions to receive the education, and all girls in pads conditions, regardless of menstrual status, to receive the two deliveries of AFRIpads. Interventions were delivered by the partner NGO. AFRIpad delivery was problematic. After the conclusion of the trial it was revealed that pads may only have been delivered to girls who reported that they had reached menarche; a very small proportion at baseline. The second pad delivery was found to be delayed from the initial intended mid-point (12 months after the first delivery), to March 2014. The names of girls receiving pads were recorded. These lists were manually matched to participants to estimate which girls in the trial received pads. This number was very low. Reports from local research assistants, however, suggested pads were left with head teachers at the schools for additional distribution. Additionally, many more pads were reported to be distributed than the number of girls listed as having received them. Thus it is highly likely that more girls received pads than those listed.

The education sessions were only run once in each school, meaning girls absent on the day of the sessions missed them. Attendance was taken and, on average, only half of all girls included in the trial were present. It is possible some names were missed, recorded incorrectly, or had been changed as part of religious practices, and so were unable to be matched to study participants. Thus, the per-protocol and intention-to-treat analysis are likely to capture more girls who did receive the interventions than displayed in the study flowchart. Nevertheless, poor fidelity was a significant issue in the trial.

### Measures

#### School attendance

As in the pilot study [26] and similar studies of menstrual hygiene management interventions,[25, 27] attendance was selected as the primary outcome. In contrast to measures such as performance, attendance is comparable across sites and other trials.

Baseline attendance: Baseline school attendance was collected prior to quasi-randomisation by copying school attendance records for two full school terms (Term 1 and 2 of 2012). Teachers received training from the partner NGO to improve the accuracy of school records. Local research assistants from the partner NGO took copies of these records each fortnight.

Follow-up attendance: The attendance data collection strategy was modified for follow-up in light of budget considerations and to further improve accuracy. Research assistants from the partner NGO conducted attendance data collection for a single week (week 4) across the three follow-up terms, with research assistants calling attendance rather than copying school registers. This was collapsed across terms 1, 2 and 3 of 2014 to form a continuous follow-up measure. Due to the change in procedure, only corresponding weeks in the baseline data (weeks 3–6 of Terms 1 and 2) were used to be comparable to the week 4 attendance collected at follow-up. Site visits and reports from local research assistants noted that school attendance varies greatly over the term due to school-fee demands and agricultural practices.

Drop-out: Throughout baseline and follow-up, if girls were absent for a full weeks’ recordings, research assistants asked other students and teachers to report if girls were simply absent from school that week or if they had dropped out of school or transferred to a new school. Student and teacher reports were checked again at subsequent data collection weeks and notes on girls’ whereabouts recorded. In instances of drop-out or transfer, research assistants recorded the name of the school the student had transferred to, or the reason a girl had dropped out of school (e.g., death in the family, pregnancy, marriage). Girls’ whereabouts were corroborated by those reached at the time of the drop-out survey, and were markedly consistent across repeated collections. Further, extensive efforts were undertaken in data cleaning phases to ensure only girls that had truly left the study schools, rather than those absent on the days assessed, were considered drop-outs.

#### Survey

Surveys captured socio-demographics, menstrual practices, and psychosocial outcomes.

Surveys were administered at baseline, mid-point, and conclusion of the study. Trained local research assistants from the partner NGO verbally administered the surveys in the local language (Lusoga) and recorded answers on iPads (using the program SurveyDeck) into an English copy of the questionnaire. Each question was required to be answered (or marked as ‘no answer’) by research assistants before it was possible to proceed to the next question, ensuring there was no accidental missing data. Survey responses were then uploaded from SurveyDeck to online collection tool, SurveyGizmo. For the final round of surveys, SurveyGizmo was available for offline use so answers were recorded on iPads directly into SurveyGizmo.

The trial employed a Solomon 4-group design, surveying only a computer randomised 25% subset (n = 281) of participants at baseline. This design was employed to reduce the bias introduced to trials by researcher interference and repeated measurement.[40, 41] In the context of the present study, there was significant concern that repeated questioning on menstrual issues would lead to additional education being provided, causing contamination. The mid-point assessment sought to follow-up only the subset of girls surveyed at baseline. However, response rates were poor and outcomes at mid-point are not reported. Survey of the full sample was attempted at the conclusion of the trial, including girls who had dropped out of school.

Menstrual practices. Girls self-reported if they had reached menarche. To assess knowledge of menstruation, interviewers asked girls to describe to them what menstruation was. Girls were considered to have passed if they revealed knowledge that menstruation was a biological process linked to reproduction, with blood lost through the vagina. Interviewers asked girls to elaborate when necessary to judge their knowledge. Additional questions captured menstrual practices. These included questions about current absorbent use, disposal, changing and washing absorbents, as well as bathing practices and girls’ access to water, soap and underwear. The survey also included items capturing how girls obtained menstrual absorbents (e.g., from parents, purchased themselves).

Psychosocial outcomes. The self-report version of the Strengths and Difficulties Questionnaire (SDQ; [42]) was used to assess overall psychosocial wellbeing. This questionnaire is a brief screening tool typically used to identify child mental health problems. The questionnaire consists of 25 items scored on a 3-point Likert scale from 0 ‘not true’ to 2 ‘true’, with a midpoint of 1 ‘somewhat true’. Questions form five subscales; hyperactivity, conduct problems, emotional problems, peer problems and prosocial behaviour, with the summative total difficulties score used in the present study. The total difficulties score includes the hyperactivity, conduct, emotional and peer problems scales summed and has a range of 0–40. For 4-17-year-olds, current 4-band categorisation based on a UK survey population are; average (0–14), slightly raised (15–17), high (18–19), very high (20–40) (see http://www.sdqinfo.com/d0.html). The SDQ has been well validated and broadly used in both high-income contexts as well as in low and middle income countries (e.g., [43]). The questionnaire has been translated into more than 75 languages.

Wellbeing in relation to menstruation was assessed through a series of single-item questions seeking to capture shame and insecurity associated with menstruation (e.g., *“During your MP*, *do you feel ashamed? Or do you feel about the same as when you are not on your MP?”*). Girls indicated if they felt more ashamed or insecure during menstruation or if they felt the same as usual.

### Analysis

Analyses were conducted using SPSS 22 [44] and Stata 14.0 [45].

Participant characteristics. Descriptive statistics were used to assess demographic characteristics. Differences in baseline demographics, psychosocial functioning, and menstrual practices across conditions were examined using the appropriate tests (see results reported). Chi-square comparisons and one-way analysis of variance were used to assess the proportion of girls who dropped out of the study for differing reasons.

Primary outcome at baseline: school attendance. Baseline school attendance was compared across conditions using a one-way analysis of variance. Attendance varied significantly, thus, subsequent analyses needed to account for baseline attendance and attendance change scores were used as the primary outcome.

Primary outcome: effect of drop-out. Variation in drop-out and reasons for drop-out across intervention conditions were assessed through one-way analysis of variance and independent samples t-tests. In addition, differences in attendance at baseline between those who dropped out of the study and those who were retained were assessed using one-way analysis of variance and independent samples t-tests.

There was considerable, and unanticipated, drop-out over the study. Due to the high proportion and reasons for drop-out, imputation would be highly unreliable and open to bias. It would have been inaccurate to assume data were missing-at-random, further it would not be theoretically justifiable to use multiple imputation for attendance scores for girls who had dropped out of school. Thus, per-protocol analysis is presented as the main analysis for the paper. Intention-to-treat analysis, described below, using two different imputation strategies is also provided.

Intervention effectiveness: primary outcome. The main study analysis was a per-protocol analysis conducted on the sample of all girls who were quasi-randomised to conditions and were still in school at the start of the attendance follow-up period. This excluded girls who had dropped out of the study schools between baseline and follow-up as there were no attendance data for these participants. Independent-samples t-tests were used to compare girls in any intervention condition to those in the control condition. One-way analysis of variance assessed differences between all four conditions, with follow-up t-tests with Bonferroni adjustment for familywise error assessing differences between the conditions.

Robustness checks. The analytic strategies outlined above did not take account of the clustered nature of the data. Whilst the preferred method of analysis would have been a multilevel model, capturing both the data clusters and individual participant variation, the very small number of clusters meant this analysis would have been inappropriate. Intra-cluster correlation coefficients were calculated to estimate the impact of clustering. In addition, the robustness of findings to adjustment for clustering was assessed through analysis of variance conducted at the cluster level (n = 8). Secondly, linear regressions with cluster-adjusted standard errors were estimated. However, these standard methods for bias correction from clustering are problematic in the context of small numbers of clusters [46] and the results were re-estimated accounting for small cluster numbers [47] as a robustness check.

Intention-to-treat analyses. Intention-to-treat analyses were conducted on the full sample of 1008 participants allocated to a condition. The large amount and nature of the missing data meant multiple imputation methods, which assume data is missing-at-random, were inappropriate. Instead, data were imputed using a realistic best case and a worst case scenario for girls’ school attendance. In the best-case, baseline observations were carried forward for missing follow-up data. In the worst-case imputation, it was assumed that girls with missing data attended school 0 days (0%). The high proportion (42.5%) and non-random nature of the missing data means these estimates are likely to be more biased than the per-protocol analysis. Nevertheless, the best-case and worst-case imputation provides a measure of the impact of missingness on the paper’s conclusions. After imputation, one-way analysis of variance was used to compare change scores between those who received any intervention and controls, and across all four conditions. Further, regressions with adjustment for the small number of clusters were undertaken as a further check of the robustness of these results to clustering.

Intervention effects for menstruating, compared to non-menstruating girls. At the conclusion of the trial, the follow-up survey indicated that less than half of the sample had reached menarche. The provision of sanitary products and education about menstruation should only benefit the school attendance of those who had started menstruating. Thus, follow-up analyses were conducted to test the impact of the intervention on girls who self-reported that they had started menstruating, and those who had not. One-way analysis of variance and independent samples t-tests were conducted on menstruating and non-menstruating sub-groups. These analyses could only be conducted for the smaller sub-sample of girls who completed the final survey (see study flow chart).

Intervention effectiveness: secondary outcomes. Psychosocial outcomes were compared between the conditions using chi-squared tests and one-way analyses of variance. These were compared between the groups at follow-up as there were no differences identified at baseline, and only a 25% sub-set of the sample was surveyed at baseline.

### Ethics

Ethical approval for the study was received from the Social Science & Humanities Inter-Divisional Research Ethics Committee at the University of Oxford (Ref No.: SSD/CUREC1/11-056), the AIDS Support Organisation (TASO) Institutional Review Committee Uganda (TASOIRC/022/14-UG-IRC-009), and the University of Illinois (#12236).

## Results

### Participant flow chart

**Fig 1:**
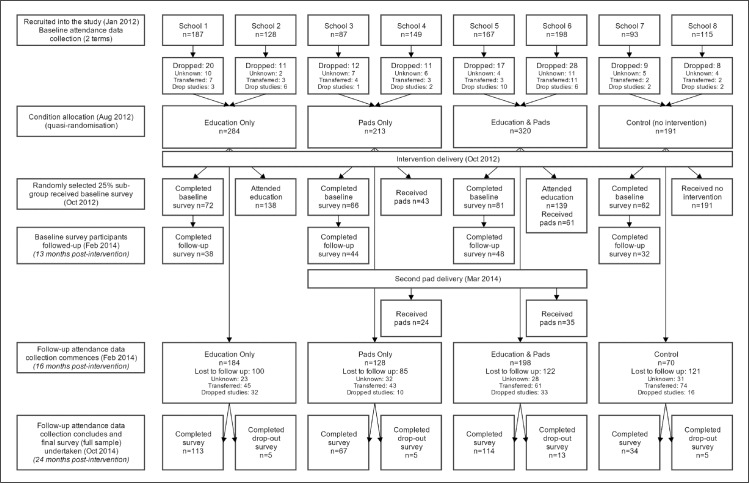
Participant flow chart.

A total of 1124 girls were recruited into the study. By the conclusion of the two-term baseline attendance data collection, 116 girls had dropped out of the schools (reasons included dropping out of school, transferring to another school, or leaving the school for an unknown reason), leaving a sample of 1008 girls across the eight schools when quasi-randomisation occurred. The participant flow chart is displayed in Fig 1. As noted in methods, the reported number of participants receiving the intervention are conservative.

**Fig 2:**
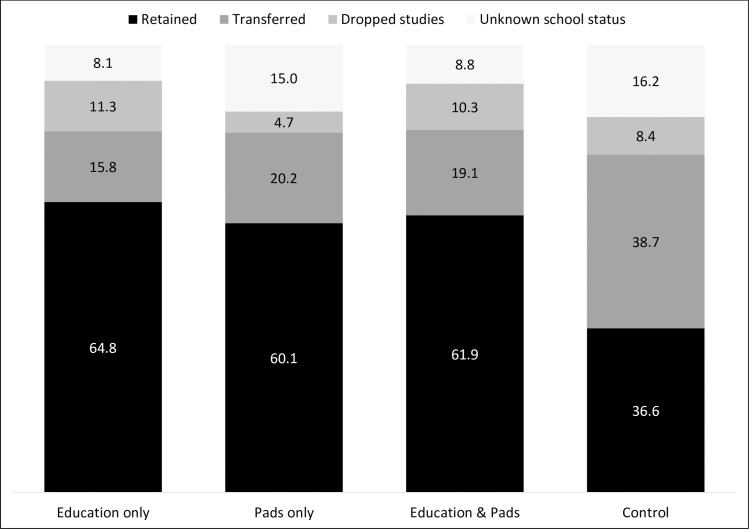
School status at follow-up according to condition (%) (n = 1008)

Participant drop-out over the study period was substantial. Consistent with low secondary school enrolment, many girls dropped out of school over the trial. Additionally, the population of girls in rural Uganda was highly mobile and many girls transferred to other schools during the trial period. At follow-up, 580 girls remained in school and 428 had dropped out of the study, a retention rate of 57.5%. Based on the reports of students and teachers, of participants lost to follow-up; 91 had dropped out of school, 223 had transferred to a new school, and 114 had unknown school enrolment status (95 were stated to have moved to a new town with unclear school enrolment, and 19 girls were no longer in school but no more information was available). A breakdown of girls’ school status across conditions at follow-up is displayed in Table 1 and Fig 2.

**Table 1:** School status at follow-up according to condition (n = 1008).

	Education only	Pads only	Education & Pads	Control
	% (N)	% (N)	% (N)	% (N)
**Retained**	64.8 (184)	60.1 (128)	61.9 (198)	36.6 (70)
**Transferred to a new school**	15.8 (45)	20.2 (43)	19.1 (61)	38.7 (74)
**Dropped studies**	11.3 (32)	4.7 (10)	10.3 (33)	8.4 (16)
**Unknown school status (moved to another town, or unknown status)**	8.1 (23)	15.0 (32)	8.8 (28)	16.2 (31)

Sites differed significantly in retention rates χ^2^(3) = 43.26, *p* < .001, with girls in the control condition far less likely to be retained in the study (36.6%) than in any intervention condition (62.4%), χ^2^(1) = 41.05, *p* < .001. This difference was driven by the number of transfers out of the control schools, with girls in the control condition significantly more likely to transfer (38.7%) than those in intervention schools (18.2%), χ^2^(1) = 36.60, *p* < .001. Girls in the control condition were no more likely to drop-out of school than those in intervention conditions (8.4% vs 9.2%, respectively), χ^2^(1) = 0.04, *p* = .842, and were significantly less likely to have an unknown school status (16.2 vs 10.2%, respectively), χ^2^(1) = 5.10, *p* = .024.

### Participant characteristics

In line with the Solomon 4-group design, a random 25% sub-sample completed the baseline survey. Table 2 displays the sample’s characteristics by intervention condition. Girls who completed the baseline survey reported being between 7 and 18 years of age (*M =* 11.44, *SD =* 1.71). Girls in the pads only schools were, on average, older than those in the control condition (*p* = .012). There were no significant differences according to school grade, repeating the current school grade, or being at the same school the preceding year. There was significant variation in the amount of time girls estimated it took to walk from home to school across the conditions χ(6) = 17.45, *p* = .008. With over half the girls in the pads only and control conditions reporting it took them less than 30 minutes, in contrast to education only and education and pads conditions where almost one third of girls reported that it took longer than one hour. The mean SDQ total difficulties score was high for girls across all conditions (*M* = 19.50, *SD =* 5.84), but did not differ significantly between conditions. Scores were in the high categorisation range based on children (aged 4–17) in the UK.[42] Although it should be noted that scores on the pro-social scale were also very high, but in the positive direction (*M* = 9.26, *SD =* 1.13).

**Table 2:** Baseline characteristics across intervention conditions in the 25% subsample (n = 281).

	Education only (72)	Pads only (66)	Education & pads (81)	Control (62)
	% (N)	%(N)	%(N)	%(N)
Age (self-report) (*M/SD*)*	11.68 (1.71)	11.89	11.20	10.97
**Grade**				
P3	37.5 (27)	28.8 (19)	30.9 (25)	43.5 (27)
P4	31.9 (23)	31.8 (21)	34.6 (28)	25.8 (16)
P5	30.6 (22)	39.4 (26)	34.6 (28)	30.6 (19)
**Are you repeating this grade?**				
Yes	27.8 (20)	42.4 (28)	25.9 (21)	29.0 (18)
No	72.2 (52)	57.6 (38)	74.1 (60)	71.0 (44)
**Did you go to the same school last year?**				
Yes	72.2 (52)	78.8 (52)	74.1 (60)	85.5 (53)
No	27.8 (20)	21.2 (14)	25.9 (21)	14.5 (9)
How long does it take you to walk to school?**				
Less than 30 mins	37.5 (27)	57.1 (36)	37.0 (30)	51.6 (32)
30 mins to 1 hour	30.6 (22)	25.4 (16)	35.8 (29)	40.3 (25)
1 hour or more	31.9 (23)	17.5 (11)	27.2 (22)	8.1 (5)
**Strengths & Difficulties Questionnaire Total Score (0–40)**	19.96 (6.49)	19.55 (5.65)	18.67 (4.88)	20.03 (6.38)
**Menstruating girls**	19.4 (14)	21.2 (14)	17.3 (14)	25.8 (16)
Does your menstruation ever cause you to miss school^1^ (Yes)	35.7 (5)	50.0 (7)	50.0 (7)	81.3 (13)
Self-reported days missed per period *(M/SD)* *	0.93 (0.25)	1.59 (0.43)	1.60 (0.43)	2.31 (1.82)

*p < .05

**p < .01

***p < .001

^1^ of menstruating girls (N = 58)

Of girls surveyed, 61.4% knew what menstruation was, and 20.6% had started menstruating. These proportions did not differ significantly by condition. Of girls who were menstruating (n = 58), 55.2% reported knowing about menstruation before it happened to them. 87.9% used cloth as menstrual absorbent with only 3 girls reporting using sanitary pads, and 4 reporting using just underwear. Almost all girls (96.6%) had access to water at home, and 86.2% of girls reported having access to soap at home. In contrast, only 15.5% reported having access to soap at school. When asked how much of a problem it was to change their sanitary protection at school 43.9% of menstruating girls stated that it was *‘not a problem at all’*, 26.3% that it was *‘a little bit of a problem’*, and 29.8% reported it was *‘a big problem’*. Most girls (70.7%) felt more ashamed and insecure whilst menstruating than when they were not. In the sample of 58 menstruating girls, no responses significantly differed between conditions.

55.2% of girls reported that their menstruation caused them to miss school. This did not differ significantly according to condition, although proportions did vary substantially (see Table 2), in the small sample. In a similar pattern, the number of days girls reported missing from school during their menses varied across conditions, *F*(3,54) = 3.05, *p* = .036. Girls in the education only condition reported missing significantly fewer days of school than those in the control condition (*p* = .026), with no other significant differences between conditions. For the 58 menstruating girls, the self-reported mean days of school missed per menses was 1.43 days (*SD* = 1.61).

### Primary outcome at baseline: school attendance

Attendance across the baseline period varied significantly by intervention condition *F*(3,1004) = 9.923, *p* < .001. Education alone (*M =* 78.02, *SD =* 23.51) and control conditions (*M* = 76.09, *SD =* 21.50) did not significantly differ in mean attendance, but both had significantly higher attendance than pads alone (*M =* 68.36, *SD =* 26.30) or education and pads conditions (*M =* 69.35, *SD =* 25.65),(*p* < .05). Education and pads, and pads alone conditions did not significantly differ in baseline attendance. Some impact of clustering was identified. Intra-cluster correlation coefficient (ICC) was calculated to be 0.026 which represents a small impact of clustering.[35]

### Primary outcome: effect of drop-out

Baseline attendance significantly differed between those who were retained or dropped out of the study F(3,1004) = 2.82, *p* = .038. However post-hoc tests revealed only those who had an unknown school enrolment status at follow-up had significantly lower baseline attendance (*M =* 67.00, *SD =* 24.79) than those who were retained at follow-up (*M =* 74.29; *SD =* 24.43), *p* = .025, with no other significant differences amongst the groups. However, these results varied when drop-outs were assessed within each condition. For those in the control schools, girls who dropped out of the study (for any reason) had significantly lower baseline attendance (*M* = 72.23, *SD* = 23.26) than those who were retained at follow-up (*M =* 82.78, *SD =* 16.12), *t*(182.83) = -3.69, *p* < .001. Similarly for girls in the education schools drop-outs had significantly lower attendance at baseline (*M* = 73.94, *SD* = 24.44) than those who were retained in the study (*M* = 80.24, *SD* = 22.76), *t*(191.36) = -2.12,*p* = .035. In contrast, for the pads only schools drop-outs did not have significantly lower attendance (*M* = 71.13, *SD =* 25.62) than those who were retained (*M* = 66.53, *SD* = 26.68), *t*(185.09) = 1.26, *p* = .208. The same was true in the education & pads schools where there was no difference in baseline attendance between drop-outs (*M =* 67.05, *SD* = 27.00) and those remaining the study (*M* = 70.77, *SD* = 24.74), *t*(239.28) = -1.24, *p* = 217. When compared across all reasons for drop-out only those in the control condition were found to vary in their baseline attendance F(3,187) = 4.75, *p* = .003, with post-hoc comparisons revealing both those who transferred from the control school, and those with unknown school status at the start of the follow-up period had significantly lower baseline attendance, (*p* = .006 and *p* = .039, respectively).

The above findings have some implications for interpreting the trial findings. The main trial analyses (per-protocol) were conducted including girls who were still attending the study schools at follow-up. Since girls with lower attendance girls were significantly more likely to drop-out of the control and education only conditions, it is likely that drop-outs would have *reduced* the average attendance in the control group, increasing the disparity between the intervention and control conditions. Thus the results of the per-protocol analysis may be conservative.

### Intervention effectiveness: school attendance

Per-protocol analysis of girls retained in the sample at follow-up was undertaken as the primary assessment of interventions’ effectiveness on school attendance (n = 580). Table 3 reports the mean percentage attendance at baseline, follow-up, and the percentage change between baseline and follow up for girls in the control condition, intervention conditions, and a pooled total for those in any intervention condition.

**Table 3:** Mean percentage attendance at baseline, follow-up and change scores across conditions (n = 580).

		Baseline %	Follow-up %	Change %
	N	*M* (*SD*)	*M* (*SD*)	*M* (*SD*)
**Education Only**	184	80.24 (22.76)	76.56 (27.47)	-3.68 (28.07)
**Pads Only**	128	66.53 (26.68)	65.63 (29.78)	-0.90 (35.43)
**Education & Pads**	198	70.77 (24.75)	64.31 (26.71)	-6.46 (32.82)
**Any intervention**	510	73.12 (25.15)	69.06 (28.30)	-4.06 (31.90)
**Control**	70	82.78 (16.12)	61.62 (29.93)	-21.16 (33.35)

**Fig 3:**
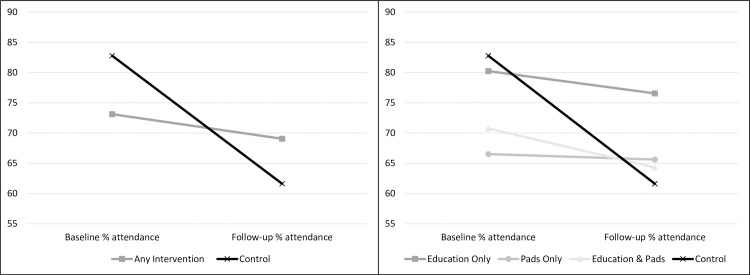
Mean percentage attendance at baseline and follow-up for all interventions combined, and each condition, compared to control. Presented for the sample of girls retained in schools at follow-up (n = 580).

Change scores differed significantly between those in the control (-21.16%) and those in an intervention condition (-4.06%), *t*(87.25) = -4.04, *p* < .001. This represented a moderate effect of any intervention in comparison to control *d* = 0.52 (95%CI 0.26–0.77). Attendance worsened across all conditions, however, in the intervention groups this effect was mitigated by 17.1% (95%CI: 8.7–25.5) (see Fig 3). This represents an extra 2.5 days in school in the 15-day (3 week) follow-up assessment period that would have otherwise been lost.

Intervention effectiveness was then compared between the four conditions. There was a significant difference amongst the groups, *F*(3,576) = 6.63, *p* < .001, η^2^ = .033. Follow-up comparisons with Bonferroni adjustment for familywise error revealed no significant differences between any of the intervention groups (*p*>.05). However, comparing the control group to each intervention, there was a significantly greater drop in attendance for the control compared to the education only (*p* = .001), pads only (*p* < .001) and education and pads (*p* = .006) conditions. This effect is presented in Fig 3.

As noted in Methods, this comparison does not take account of the clustered nature of the data. The intra-cluster correlation coefficient (ICC) for follow-up attendance data was 0.058, which represents a small impact of clustering.[48]

#### Robustness checks

While above analyses provide easily interpretable estimates of intervention effects, they do not adjust for clustering. Additional analyses were undertaken with adjustment for clustering, bearing in mind the small number of clusters.

Ordinary least squares regression with adjustment for clustering compared the three intervention conditions to the control. Again there was an overall effect of condition F(3,7) = 14.00, *p* = .002, *R*^*2*^ = 0.03. Each intervention condition; education alone (*b* = 17.48, *SE* = 4.69, 95%CI = 6.40–28.56), pads alone (*b* = 20.26, *SE* = 3.18, 95%CI = 12.75–27.78), and education and pads (*b* = 14.70, *SE* = 3.58, 95%CI = 6.23–23.17) significantly differed from the control. With adjustment for clustering, considering the small number of clusters, there was also an overall effect of the intervention. Each intervention condition; education alone (*b* = 17.48, *SE* = 4.69, 95%CI = 8.29–26.67), pads alone (*b* = 20.26, *SE =* 3.18, 95%CI = 14.02–26.49), and education and pads (*b =* 14.70, *SE* = 3.58, 95%CI = 7.68–21.73) differed significantly from the control. Similarly, adjustment for clustering demonstrated the robustness of results where any intervention was compared to the control. Results were corroborated with ordinary least squares regression with cluster adjustment, where there remained an effect of any intervention (*b* = 17.10, *SE* = 3.45, 95%CI = 8.95–25.25) compared to the control F(1,7) = 24.64, *p =* .002, *R*^*2*^ = 0.03, and in regression with adjustment for clustering accounting for the small number of clusters (*b* = 17.10, *SE* = 3.45, 95%CI = 10.34–23.86).

One-way analysis of variance at the school/cluster level (n = 8) found an overall significant difference between conditions, F(3,4) = 6.80, *p* = .048, however, follow-up tests with Bonferroni adjustment were not significant. This is to be expected in light of the lack of power to detect effects at the cluster level only (n = 8). All intervention conditions, education alone (p = .164), pads alone (*p* = .080) and pads and education (*p* = .246), trended toward a significant difference from the control condition.

### Intention-to-treat analyses

Worst case imputation. When 0% attendance was imputed for all girls who had dropped out of the study the mean percentage attendance at follow-up decreased dramatically (Table 4). Consistent with per-protocol analyses there was a significantly greater drop in attendance for girls in the control condition than those in the intervention condition *t*(328.52) = -7.98, *p* < .001. This was a moderate effect *d* = 0.62 (95%CI 0.46–0.78). Similarly, a one-way ANOVA identified a significant difference between group change scores when individual interventions were considered F(3,1004) = 17.09, *p* < .001, η^2^ = 0.05. Post hoc tests found no significant differences amongst the intervention conditions, but all intervention conditions had a significantly smaller drop in attendance than those in the control condition (*p* < .001 for all analyses). Regression with adjustment for the small number of clusters demonstrated the robustness of results to clustering, finding when compared to the control, the change in attendance percent was mitigated by 5.2% in the intervention conditions (*b* = 5.22, *SE* = 1.83, 95%CI = 1.63–8.81).

**Table 4:** Mean percentage attendance at baseline, follow-up and change scores across conditions for best and worst case imputations (n = 1008).

			Worst case imputation		Best case imputation	
		Baseline %	Follow-up %	Change %	Follow-up %	Change %
	N	*M* (*SD*)	*M* (*SD*)	*M* (*SD*)	*M* (*SD*)	*M* (*SD*)
**Education Only**	284	78.02 (23.51)	46.60 (42.78)	-28.42 (42.98)	75.64 (26.43)	-2.39 (22.64)
**Pads Only**	213	68.36 (26.30)	39.44 (39.61)	-28.93 (46.91)	68.82 (28.26)	-0.54 (27.43)
**Education & Pads**	320	69.35 (25.65)	39.79 (37.67)	-29.56 (42.55)	65.35 (26.81)	-4.00 (25.98)
**Any intervention**	817	72.11 (25.45)	43.11 (40.24)	-29.00 (43.83)	69.57 (27.41)	-2.54 (25.28)
**Control**	191	76.09 (21.50)	22.58 (34.81)	-53.51 (36.80)	68.33 (26.33)	-7.76 (22.55)

Best case imputation. An intention to treat analysis with last observations (baseline attendance) carried forward was also conducted. Consistent with main analyses, there was a significantly greater drop in attendance for girls in the control condition than those in the intervention condition *t*(311.80) = -2.81, *p* = .005, *d* = 0.21 (95%CI 0.05–0.37). When intervention groups were compared separately there remained a significant difference in change scores amongst the groups F(3,1004) = 3.13, *p* = .025, η^2^ = 0.01, however, post hoc tests found only the pads alone schools had a significantly smaller drop in attendance than those in the control schools (*p* = .021). These results were also robust to the impact of clustering with any intervention mitigating the drop in percent attendance by 24.5% (*b* = 24.51, *SE* = 2.25, 95%CI = 20.10–28.91).

Both scenarios indicate a more pronounced decrease in the control group attendance compared to the intervention groups, consistent with the pattern of results for the per-protocol analysis. This suggests the positive effect of the interventions represents a real difference, but that the precise amount of attendance saved by the interventions may vary. Whilst per-protocol analysis observed a difference of 17.1% (95%CI: 8.7–25.5) between interventions and control, conservative imputation estimated 5.2%, and worst-case imputation estimated 24.5%. These results were robust to adjustment for clustering.

### Outcomes according to menstrual status

As noted in methods, the trial included girls both pre- and post-menarche with the aim of delivering the intervention before menstruation commenced. However, fewer girls had reached menarche than anticipated in the final survey (although those who had dropped out of school and were not reached for survey are likely to be older and to have reached menarche so figures in the final survey may be an underestimate). Thus, additional comparisons were undertaken for girls who completed the final survey (n = 356) and reported that they had reached menarche (n = 164), and those who had not (n = 192). These analyses also served as an additional check in light of the small number of clusters in the trial. If the same pattern of results was observed for girls pre- and post-menarche it may have suggested that unidentified cluster-level factors may have been driving identified effects. Table 5 displays the percentage attendance and change scores for girls in each condition, or who received any intervention, according to whether they self-reported that they had or had not reached menarche at the time of the final survey.

**Table 5:** Mean percentage attendance at baseline, follow-up and change scores across conditions for menstruating and non-menstruating girls at follow-up (n = 356).

		Baseline %	Follow-up %	Change %
	N	*M* (*SD*)	*M* (*SD*)	*M* (*SD*)
Girls post-menarche at follow-up (N = 164)				
**Education Only**	40	84.29 (19.14)	82.83 (22.23)	-1.46 (16.55)
**Pads Only**	40	74.10 (23.83)	79.33 (21.18)	5.23 (29.48)
**Education & Pads**	70	72.38 (23.02)	72.19 (22.81)	-0.19 (27.66)
**Any Intervention**	150	76.02 (22.70)	76.93 (22.57)	0.92 (25.70)
**Control**	14	85.71 (13.85)	61.90 (27.10)	-23.81 (34.82)
Girls pre-menarche at follow-up (N = 192)				
**Education Only**	78	83.99 (19.85)	87.95 (13.89)	3.96 (21.64)
**Pads Only**	32	65.38 (26.14)	80.21 (16.63)	14.82 (28.24)
**Education & Pads**	57	67.88 (27.66)	73.22 (19.78)	5.34 (30.56)
**Any Intervention**	167	74.93 (25.33)	81.44 (17.79)	6.51 (26.42)
**Control**	25	85.03 (13.80)	85.07 (13.09)	0.04 (18.14)

**Fig 4:**
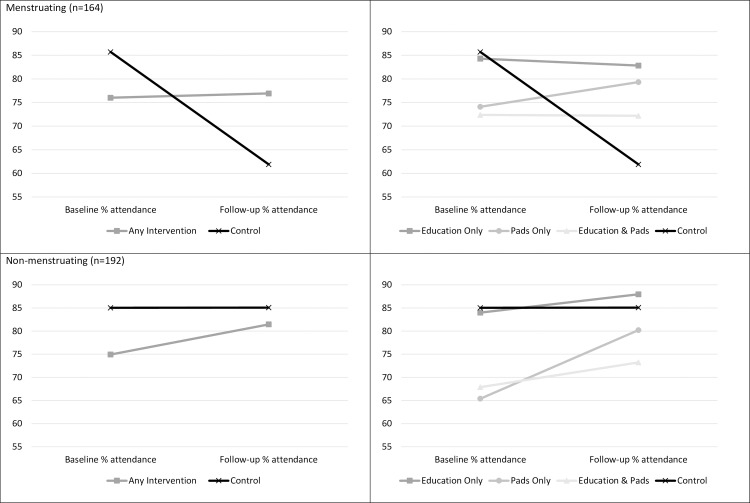
Mean baseline and follow-up percentage attendance according to girls’ menstrual status at study conclusion (n = 356)

For girls who had commenced menstruation by the time of the final survey, receiving any intervention was associated with a significant difference in attendance change score, *t*(14.35) = -2.59, *p* = .021, *d* = 0.93 (95%CI 0.37–1.49), a mean difference of 24.7% (95%CI 10.07–39.38). When distinguishing between the interventions there was an overall difference in change scores, F(3,160) = 4.19, *p* = .007, η^2^ = 0.07, and follow-up comparisons revealed no significant differences between the intervention groups. But that education (*p* = .045), pads (*p* = .003) and education and pads (*p =* .017) conditions all experienced a significantly smaller drop in attendance than the control condition. For girls who had not yet reached menarche at the time of the final survey, receiving any intervention was not associated with a significant difference in the changed attendance percentage between baseline and follow-up, *t*(41.06) = -1.55, *p* = .128, *d* = 0.25 (95%CI -0.17–0.67). There was also no significant difference in change scores when distinguishing between the intervention types F(3,188) = 1.92, *p* = .128, η^2^ = 0.03. Interestingly, change scores revealed a slight increase in attendance for these girls. Results are also shown in Fig 4.

### Intervention effectiveness: psychosocial wellbeing

A total of 356 girls from the trial completed a final survey. Of these, 328 completed the full follow-up survey, and 28 girls who had dropped out of school completed a shorter ‘drop-out’ version of the survey. The mean self-reported age of this sample was 13.23 (SD = 1.43). 46.1% of girls surveyed at the end of the study had started menstruating. Since psychosocial wellbeing constructs were balanced across the groups at baseline, follow-up scores were compared. Table 6 displays the self-reported SDQ total score, and feelings of shame and insecurity during menstruation across the four conditions. Of girls who completed the follow-up surveys, a higher proportion were menstruating in the pads only and education and pads schools, compared to the education only and control schools χ(1) = 14.57, *p* < .001. For girls who were menstruating, there were no differences across the conditions on any outcome, including feelings of shame or insecurity during menstruation.

**Table 6:** Psychosocial wellbeing across intervention conditions (n = 356).

	Education only (118)	Pads only (72)	Education & pads (127)	Control (39)
	% (N)	%(N)	%(N)	%(N)
Accurate knowledge of menstruation^1^	58.5 (55)	71.2 (42)	67.7 (65)	63.3 (19)
Strengths & Difficulties Questionnaire Total Score^2^ (0–40) *M(SD)*	18.41 (4.75)	19.39 (5.39)	18.61 (4.90)	17.18 (5.62)
Menstruating girls^3^***	33.9 (40)	55.6 (40)	55.1 (70)	35.9 (14)
Feels more ashamed during menstruation^4^	77.5 (31)	65.0 (26)	61.4 (43)	64.3 (9)
Feels more insecure during menstruation^4^	75.0 (30)	65.0 (26)	68.6 (48)	71.4 (10)
Does your menstruation ever cause you to miss school?^5^ (Yes)	20.0 (7)	8.8 (3)	23.6 (13)	11.1 (1)
Self-reported days missed per period^5^ *M(SD)*	0.37 (0.88)	0.24 (0.86)	0.38 (0.81)	0.33 (1.00)

*p < .05

**p < .01

***p < .001

^1^of girls who completed the full follow-up survey, 12 girls did not answer, for 35 there was an ipad error (n = 279)

^2^of girls completing full follow-up survey, 2 girls did not provide responses (n = 326)

^3^of girls completing full follow-up or drop-out survey (n = 356)

^4^of menstruating girls who completed either full follow-up or drop-out survey (n = 164)

^5^of menstruating girls who completed full follow-up survey (n = 133)

## Discussion

### Main study findings

This trial assessed the effectiveness of providing reusable sanitary pads or puberty education for improving girls’ school attendance in rural Uganda. Consistent with the pilot study in Ghana, the trial found both education and reusable sanitary pads to be equally effective in improving school attendance compared to no-intervention. For all girls still in the study schools at follow-up, attendance had dropped substantially from the baseline. However, those in the control schools had a 17.1% (95%CI: 8.7–25.5) greater drop in attendance than those who received the interventions in per-protocol analyses. This represents an extra 2.5 days in school in the 15-day (3 week) follow-up assessment period that would have otherwise been lost. The moderate effect size found in this study (*d* = 0.52 95%CI 0.26–0.77) was comparable to the pooled standardised mean difference revealed by meta-analysis of the two pilot trials of sanitary pad and education provision to date (*d* = 0.49; [26, 27, 34]). In this trial, the provision of education or reusable pads alone, and these interventions combined, were found to have similar effectiveness in improving attendance. For policy makers, this suggests that even in absence of resources to provide sanitary pads, the inclusion of adequate and gender-sensitive puberty education in school curriculum could improve attendance and target gender inequalities. Regressions with adjustment for clustering demonstrated the robustness of the findings. The trial suffered from significant drop-out (42.5%). Only a sub-set of girls were surveyed at baseline, precluding multiple imputation based on this survey. Further, the nature of the participant drop-out meant that imputed attendance scores would be misrepresentative. Thus two intention-to-treat analyses with different imputation strategies were undertaken. The first, or worst-case scenario, assumed all drop-outs had 0% school attendance, while the second, best-case scenario, presented a highly conservative estimate where baseline attendance scores were carried forward for all drop-outs. In both scenarios, the pattern of results observed in the per-protocol analyses were replicated and the interventions were found to have a significant positive effect on attendance (5.2% in the best-case, and 24.5% in the worst-case estimates).

The frequency at which girls dropped out of school or transferred to another school was unanticipated. While school absences attributable to menstrual management may be an additional factor involved in drop-out, menstruation alone was not hypothesised to lead to drop-out and drop-out was not considered as an outcome variable in the study. The distribution of participant drop-out for various reasons was similar across sites. There were no differences in the rate dropping out of school or moving away, however the proportion of girls transferring to another school during the study was substantially higher in the control condition. Follow-up assessment revealed this was spread across both control schools. There were no patterns in the recorded destination schools for transfer. Interventions may have been protective against school transfer, as improved attendance may have meant girls were better equipped to pass school exams and to progress through the school at the expected rate, reducing motivation to transfer. It is also possible that the presence of the partner NGO in those schools delivering pads or education provided extra incentive to stay, although the additional attention should only have occurred very early in the study and should not have been sufficient to influence school choice. Given the small number of clusters it is also possible an unmeasured factor influenced the substantial transfer from the control schools.

The study included girls in upper primary school (ages 10–18, with a mean of 11.44 at baseline) and sought to intervene before menarche. Only 20% of the sample had commenced menstruating at the time of intervention. Despite age at menarche estimates of 12–14,[49, 50] only 46% of girls self-reported having commenced menstruation at the conclusion of the trial. This may reflect the variability of menarche with environmental factors such as nutrition, and means the intervention was well-timed to educate girls and provide products prior to menarche, but that the follow-up period was not long enough for most girls to have started menstruating. It is also likely that girls who had been lost to follow-up were older and more likely to have reached menarche, thus the survey figure is an underestimate. The measured effect of the intervention is therefore likely to be conservative as the provision of pads or education should make little difference to girls prior to menarche.

To provide an estimate of the intervention effectiveness amongst girls who had reached menarche, additional analyses were undertaken separately for menstruating and non-menstruating girls. These analyses were restricted to the smaller sample of girls who had completed the follow-up surveys. In addition to estimating the effectiveness of the interventions for those who had reported reaching menarche in the follow-up surveys, these analyses served to check for variations in attendance which may have been caused by individual school level factors. Moreover, to assess the consistency of the school attendance measures which were changed between baseline (teacher-recorded attendance) and follow-up (partner-NGO recorded attendance) and could have introduced bias. Analysis according to menstrual status supported the interpretation of the per-protocol analysis, finding a significant, 24.7% (95%CI 10.07–39.38) drop in attendance in the control conditions for girls’ post-menarche compared to the intervention conditions, which did not differ from one another. In contrast, there was no effect of the interventions, and no drop in attendance amongst girls who reported not yet menstruating. Findings support the interpretation of the study results that intervening in menstrual management was responsible for observed differences in school attendance. Further, findings support the hypothesised link between menstruation and school absence,[18, 23] although these differences may also reflect other age-related absences, such as increased obligations to paid work or household chores.

### Secondary outcomes

There were no significant differences in the psychosocial outcomes assessed across conditions at follow-up. Reductions in shame and insecurity in the conditions providing sanitary pads in the pilot study were not replicated.[22] It should be noted that only a small proportion of girls in the study completed the follow-up survey, and single item measures of shame and insecurity may have been influenced by social-desirability, particularly as the survey was administered verbally by research assistants. Similarly, the SDQ total difficulties score[42] did not differ across groups. Very high total difficulties scores were noted. These high scores may reflect the study population. Recent systematic review, including studies using the SDQ, concluded that considerable levels of mental health problems exist among children and adolescence in sub-Saharan Africa.[51] Higher scores on subscales of the SDQ have been noted in studies in the Democratic Republic of Congo [52] and refugee children in the UK,[53] although these were both in younger populations of children then the present study. Interestingly, in this study scores on the pro-social scale were also very high, but in the positive direction. This may reflect some bias towards affirmative responses, perhaps again reflective of the verbal administration of the survey. There was no difference in girls’ knowledge of menstruation at follow-up, however, the measurement of this construct was poor. During the survey girls were asked to describe menstruation and research assistants provided a pass/fail rating based on criteria that girls reported menstruation to be a biological process associated with reproduction. This menstrual knowledge measure fails to capture many important aspects of menstrual management, and may be biased based on girls’ confidence to describe the process or research assistants’ subjective assessment of the response adequacy. Improved measurement is needed in future trials.

The trial failed to assess potential harms of the interventions. The identification of menstruating girls through education and the provision of sanitary products has the potential to expose these girls’ menstrual status, leading to increased stigmatisation, teasing, or unwanted attention. To date, no studies of menstrual management interventions have assessed potential harms (see [34]), and there is an urgent need for future work to more thoroughly investigate this possibility.

### Implications for menstrual hygiene intervention and research

Results of the study support the assertion that the management of menstruation presents a gender specific barrier to education.[33] They also support the provision of menstrual absorbents and information about menstruation as effective avenues for intervention. It should be noted, however, that the effective and hygienic management of menstruation requires more than knowledge or reusable absorbents.[54] The interventions assessed in this trial did not address needs for adequate latrines, access to water for washing the body and absorbents, access to soap (beyond a small quantity provided initially with reusable pads in some conditions), access to privacy or methods for disposal. Each of these factors contributes to dignity and enables adequate menstrual management.[54, 55] As access to these other requirements for menstrual management were comparable across schools. Follow-up qualitative work to be detailed elsewhere, and reports from the field revealed that girls cleaned absorbents at home rather than at school, and that changing practices were associated with individual school cultures around latrine use rather than any variation in facilities. In taking a holistic view of girls’ needs, it is important to consider the way access to other requirements for management may further contribute to school attendance, and to psychosocial wellbeing, which was not improved by the tested interventions. Furthermore, as a reusable sanitary pad was provided in this intervention, the effectiveness of this product is contingent on the resources to wash and dry it appropriately.

Girls’ psychosocial wellbeing, dignity, comfort, and ability to manage menstruation without shame are all essential considerations in seeking to provide girls with their full right to menstrual health in low income contexts. Whilst there was only limited measurement of these outcomes in the present trial, the lack of an effect is disappointing and means that there is more work to be done for the development and evaluation of interventions to address this challenge. Increased primary epidemiological research capturing girls’ behaviours, attitudes, and barrier to menstrual hygiene, as well as improved measurement of negative effects on wellbeing or comfort would provide much needed knowledge and tools for intervention research.

Limitations of the current trial should be addressed in future studies. *A-*priori analytic strategies for high levels of participant drop-out are needed where this is expected. For girls who have dropped out of school, no attendance can be expected. These cases may zero-inflate data and fail to capture the true nature of effects. Trials with a larger number of clusters are needed to enable adequate samples for multi-level models. Full baseline survey may be necessary to adequately compare lost and retained participants, however the risks of bias associated with multiple testing or the impromptu education provided through asking questions about menstruation in studies of the effectiveness of puberty education must be considered. Menstrual diaries may allow for more specific menstrual-attendance measures but may also represent an intervention if girls had not previously recorded their cycles, and risk greater quantities of missing data where diaries are not returned.

### Contextual considerations and implementation

This paper reports on the primary quantitative outcomes of the *menstruation and the cycle of poverty* trial. The study took place in a complex environment and many factors should be considered to maximise understanding of the barriers and facilitators of girls’ schooling in rural Uganda, and lessons for future trials of sanitary pad provision or puberty education. Forthcoming qualitative and quantitative work will seek to elucidate further complexities of improving girls’ menstrual management and access to schooling. In brief, these include the role of teachers, whether or not girls used the provided pads or if they were sold to combat other barriers to schooling such as school fees, the acceptability of a reusable absorbent, and girls cleaning practices. The interaction of the intervention with the other considerations for menstrual management noted above, including how girls managed menstruation when only education was provided, disposal or the adequacy of cleaning methods for the reusable pads provided, and the role of access to water, sanitation and hygiene and privacy with regard to school latrines, are all important considerations in understanding how the interventions worked and what adaptations to the interventions may be needed if relocated to different contexts. Current results do not have sufficient explanatory power for why education alone, pads alone, and education in addition to pads did not differ in their effectiveness for improving school attendance or psychosocial outcomes.

Broader contextual considerations for this study include the roll-out of the partner NGO, Plan International’s well-known *‘Because I am a girl’* campaign (https://plan-international.org/what-we-do/because-i-am-girl) may have influenced community attitudes regarding girls’ education. The effect of the campaign should have been comparable across conditions, however, an increased emphasis on girls’ education may have impacted the self-reported psychosocial and menstrual practice findings. Girls’ reports must be interpreted in light of this program. An unanticipated contextual issue was the practice of labial elongation in the trial population. This will be discussed further in forthcoming qualitative work, however, reports from girls in the study suggested that uptake of the reusable sanitary pads may have been limited by discomfort, perhaps associated with this practice.

### Study strengths and limitations

This trial was the largest to date assessing the impact of menstrual management interventions on girls’ school attendance. The use of a clustered design prevented contamination, while allocation to the four conditions provided a contrast between the different intervention components of education and sanitary pad provision, compared to a no-intervention control. The sequential allocation of clusters to conditions alphabetically represented quasi-randomisation, potentially resulting in a higher risk of selection bias.[56] However, despite this sub-optimal randomisation in comparison to more rigorous methods such as computer randomisation, the school names in alphabetical order is unlikely to have biased allocation. There were few differences in participant characteristics between the conditions measured at baseline. The study did not adjust for slight differences in mean age and the distance walked from school as only a portion of the participants were surveyed at baseline, age is highly collinear with menstrual status, and there was no evidence to suggest the distance walked to school would have any significant interaction with the intervention. The small number of clusters limits the generalisability of findings and meant the trial was vulnerable to external factors which may have influenced any of the small number of sites. The high rate of drop-out, and inability to follow-up girls who dropped or transferred out of study schools also restricted the validity of the intention-to-treat analysis and ability to capture the pattern of attendance for the full sample.

The change in attendance data collection methods was a significant limitation of the study. Despite receiving training and repeated checks to try and ensure the accuracy of teacher-recorded attendance, changing the follow-up measure to three, single-week attendance data collections by research assistants may have introduced bias. The drop in attendance between baseline and follow-up may be a true effect, but may also reflect this change in method. The impact of the change should be the same across schools, and should not limit the interpretation of the effect sizes as demonstrated in sub-group analyses of girls pre- and post-menarche. The use of only two time points (1 week of attendance data per term for the follow-up year), means more nuanced changes in attendance over time could not be investigated (for example, if there was an initial increase in attendance following intervention delivery or if education took longer to achieve a change as was found in the pilot study). Similarly, restriction of school attendance records to weeks in the middle of the term means results may not be representative of other times when attendance may vary more substantially. It should be noted that this study assessed attendance across all days, not only attendance during menstruation. Poor follow-up survey coverage limits the study from being able to test mediating effects such as the role of changed menstrual practices which may have resulted from education interventions as sample sizes of menstruating girls who completed the follow-up survey were highly limited.

Weaknesses in intervention delivery also severely limit the reliability of results found. Although estimated, it is unclear what proportion of girls received the assigned interventions. Intervention implementation through a local NGO brought strengths to the study, providing access to the local language, age appropriate research assistants to administer surveys and rapport with local communities and schools. However, issues with delivery and long-armed management from the UK resulted in poor intervention implementation and inadequacies in participant follow-up.

## Conclusions

This cluster quasi-randomised controlled trial provides initial evidence for the effectiveness of providing reusable sanitary pads or puberty education for improving girls’ school attendance in rural Uganda. Given the limitations of the trial, and need to assess potential harms of menstrual management interventions, more research and replication is needed. Challenges identified in this trial represent important considerations for future work. The study adds to an emergent body of evidence suggesting that interventions to assist girls in better managing their menses may be effective in improving girls’ access to education and reducing gender inequality.

## Supporting Information

S1 Table CONSORT 2010 checklist of information to include when reporting a cluster randomised trial. (PDF)

S2 Table School characteristics at baseline. (PDF)

S1 File Trial protocol. Trial protocol registration (Pan African Trials Registry PACTR201503001044408)
(PDF)

## References

[1] Sperling GB, Winthrop R. What Works in Girls' Education: Evidence for the World's Best Investment: Brookings Institution Press; 2015.

[2] OECD. Closing the Gender Gap: Act Now. Paris: OECD, 2012.

[3] Barro RJ, Lee JW. A new data set of educational attainment in the world, 1950–2010. Journal of development economics. 2013;104:184–98.

[4] LeVine RA, LeVine SE, Rowe ML, Schnell-Anzola B. Maternal literacy and health behavior: a Nepalese case study. Social science & medicine. 2004;58(4):863–77. 14672599 10.1016/s0277-9536(03)00261-2

[5] Kirk J, Sommer M. Menstruation and body awareness: linking girls’ health with girls’ education. Royal Tropical Institute (KIT), Special on Gender and Health. 2006:1–22. http://www.schools.watsan.net/content/download/323/2726/

[6] Lloyd CB, Young J. New lessons: the power of educating adolescent girls: a Girls Count report on adolescent girls 2009; Population Conuncil: New York.

[7] Gakidou E, Cowling K, Lozano R, Murray CJ. Increased educational attainment and its effect on child mortality in 175 countries between 1970 and 2009: a systematic analysis. The Lancet. 2010;376(9745):959–74. 10.1016/S0140-6736(10)61257-3 20851260

[8] Montenegro CE, Patrinos HA. Comparable estimates of returns to schooling around the world. World Bank policy research working paper. 2014;(7020).

[9] UNESCO. Education for All 2000–2015: Achievements and Challenges—Education for All Global Monitoring Report 2015. Paris: UNESCO, 2015.

[10] UNESCO. Education Statistics, UNICEF Division of Policy and Practice, Statistics and Monitoring. http://www.childinfo.org/files/ESAR_Uganda.pdf (accessed September 2010): UNESCO, 2008.

[11] World Bank. Country and Lending Groups: Data. Available from http://data.worldbank.org/about/country-classifications. 2014.

[12] Uganda Demographic and Health Survey (UDHS). Uganda Bureau of Statistics: Kampala, Uganda. 2011.

[13] World Bank. By Country Data available from http://data.worldbank.org/indicator/SE.PRM.ENRR/countries. 2015.

[14] Clinton Foundation, Bill & Melinda Gates Foundation, Economist Intelligence Unit, and WORLD Policy Analysis Center. The Full Participation Report: No Ceilings. New York: Clinton Foundation, 2015.

[15] UNESCO. Global education digest: comparing education statistics across the world. 2010: Unesco Institute for Statistics 2010; Montreal, CA.

[16] Lloyd CB, Mensch BS. Marriage and childbirth as factors in dropping out from school: an analysis of DHS data from sub-Saharan Africa. Population Studies. 2008;62(1):1–13. 10.1080/00324720701810840 18278669

[17] Parkes J, Heslop J. Stop Violence Against Girls at School: A Cross-Country Analysis of Change in Ghana, Kenya and Mozambique. London/ Johannesburg, South Africa, Institute of Education, University of London/ ActionAid International 2013.

[18] Sommer M, Sutherland C, Chandra-Mouli V. Putting menarche and girls into the global population health agenda. Reproductive health. 2015;12(1):24. 25889785 10.1186/s12978-015-0009-8 PMC4396832

[19] McMahon SA, Winch PJ, Caruso BA, Obure AF, Ogutu EA, Ochari IA, et al ‘The girl with her period is the one to hang her head' Reflections on menstrual management among schoolgirls in rural Kenya. BMC international health and human rights. 2011;11(1):7. 21679414 10.1186/1472-698X-11-7 PMC3129305

[20] Sommer M, Kirk J. Menstruation is on her mind’: Girl-centered, holistic thinking for school sanitation, WASH in Schools Notes and News. International Water and Sanitation Centre 2008:4–6. Available from http://www.irc.nl/page/39859.

[21] Sommer M, Ackatia-Armah N, Connolly S, Smiles D. A comparison of the menstruation and education experiences of girls in Tanzania, Ghana, Cambodia and Ethiopia. Compare: A Journal of Comparative and International Education. 2014;1–21.

[22] Dolan CS, Ryus CR, Dopson S, Montgomery P, Scott L. A blind spot in girls’ education: menarche and its webs of exclusion in Ghana. Journal of International Development. 2013;26(5), 643–657.

[23] Tegegne TK, Sisay MM. Menstrual hygiene management and school absenteeism among female adolescent students in Northeast Ethiopia. BMC public health. 2014;14(1):1118. 25355406 10.1186/1471-2458-14-1118 PMC4232635

[24] Grant M, Lloyd C, Mensch B. Menstruation and School Absenteeism: Evidence from Rural Malawi. Comparative Education Review. 2013;57(2):260–84. 10.1086/669121 25580018 PMC4286891

[25] Oster E, Thornton R. Menstruation, sanitary products, and school attendance: Evidence from a randomized evaluation. American Economic Journal: Applied Economics. 2011:91–100.

[26] Montgomery P, Ryus CR, Dolan CS, Dopson S, Scott LM. Sanitary Pad Interventions for Girls' Education in Ghana: A Pilot Study. PloS one. 2012;7(10):e48274 10.1371/journal.pone.0048274 23118968 PMC3485220

[27] Wilson E, Reeve J, Pitt A. Education. Period. Developing an acceptable and replicable menstrual hygiene intervention. Development in Practice. 2014;24(1):63–80.

[28] Pillitteri SP. School menstrual hygiene management in Malawi: more than toilets. WaterAid. 2011.

[29] Crichton J, Okal J, Kabiru CW, Zulu EM. Emotional and Psychosocial Aspects of Menstrual Poverty in Resource-Poor Settings: A Qualitative Study of the Experiences of Adolescent Girls in an Informal Settlement in Nairobi. Health Care for Women International. 2013;34(10):891–916. 10.1080/07399332.2012.740112 23570366

[30] Sumpter C, Torondel B. A Systematic Review of the Health and Social Effects of Menstrual Hygiene Management. PloS one. 2013;8(4):e62004 10.1371/journal.pone.0062004 23637945 PMC3637379

[31] Garg R, Goyal S, Gupta S. India moves towards menstrual hygiene: subsidized sanitary napkins for rural adolescent girls—issues and challenges. Maternal and child health journal. 2012;16(4):767–74. 10.1007/s10995-011-0798-5 21505773

[32] Proctor & Gamble. Keeping girls in school: supporting girls health to promote their empowerment. 2010; Available at: http://www.pg.com/en_ZA/sustainability/social-responsibility/always-keeping-girls-in-school.shtml

[33] Sommer M, Hirsch JS, Nathanson C, Parker RG. Comfortably, Safely, and Without Shame: Defining Menstrual Hygiene Management as a Public Health Issue. American Journal of Public Health. 2015;(0):e1–e10. 10.2105/AJPH.2014.302525 PMC4463372 25973831

[34] Hennegan J, Montgomery P. Do Menstrual Hygiene Management Interventions Improve Education and Psychosocial Outcomes for Women and Girls in Low and Middle Income Countries? A Systematic Review. PloS one. 2016;11(2):e0146985 10.1371/journal.pone.0146985 26862750 PMC4749306

[35] Campbell MK, Piaggio G, Elbourne DR, Altman DG. Consort 2010 statement: extension to cluster randomised trials. 2012;345:e5661 10.1136/bmj.e5661 22951546

[36] <j/>Republic of Uganda. Kamuli District Local Government Five Year Strategic Plan, Fiscal Year 2008/09–2012/13. 2008; Available at http://s3.amazonaws.com/zanran_storage/www.coreinitiative.org/ContentPages/44444706.pdf

[37] Uganda Development Services. Primary Literacy Project. 2013; available at: http://www.ugandadev.org.uk/what-we-do/library/literacy/.

[38] Nakabugo Z. 4,000 Kamuli pupils drop out of school. The Observer. 2013; available at http://observerug/education/85-education/27374-4000-kamuli-pupils-drop-out-of-school.

[39] Scott L, Montgomery P, Stenfield L, Dolan C, Dopson S. Sanitary pad acceptability and sustainability study Oxford: University of Oxford, 2013.

[40] McCambridge J, Butor-Bhavsar K, Witton J, Elbourne D. Can research assessments themselves cause bias in behaviour change trials? A systematic review of evidence from Solomon 4-group studies. PLoS One. 2011;6(10):e25223 10.1371/journal.pone.0025223 22039407 PMC3198466

[41] McCambridge J, Witton J, Elbourne DR. Systematic review of the Hawthorne effect: new concepts are needed to study research participation effects. Journal of clinical epidemiology. 2014;67(3):267–77. 10.1016/j.jclinepi.2013.08.015 24275499 PMC3969247

[42] Goodman R, Meltzer H, Bailey V. The Strengths and Difficulties Questionnaire: a pilot study on the validity of the self-report version. European child & adolescent psychiatry. 1998;7(3):125–30. 9826298 10.1007/s007870050057

[43] Syed EU, Hussein SA, Azam SI, Khan AG. Comparison of Urdu version of Strengths and Difficulties Questionnaire (SDQ) and the Child Behaviour Check List (CBCL) amongst primary school children in Karachi. Journal of the College of Physicians and Surgeons Pakistan. 2009;19(6):375 19486578

[44] IBM Corporation. IBM SPSS Statistics for Windows, Version 22.0. Armonk, NY: IBM Corp. 2013.

[45] StataCorp. Stata Statistical Software: Release 14. College Station, TX: StataCorp LP 2015.

[46] Cameron AC, Gelbach JB, Miller DL. Bootstrap-based improvements for inference with clustered errors. The Review of Economics and Statistics. 2008;90(3):414–27.

[47] Gelbach J, Miller D. Cgmreg.ado—Robust Inference with multi-way clustering- User-written Package for Stata. 2009. Available at http://gelbach.law.upenn.edu/~gelbach/ado/cgmreg.ado [Last access, 2016 Jan 20].

[48] Chromy JR. Modeling Cluster Design Effects When Cluster Sizes Vary In JSM Proceedings, Survey Research Methods Section. Alexandria, VA: American Statistical Associationp.4323–4328. Available at http://www.amstat.org/sections/SRMS/Proceedings/

[49] Anderson SE, Dallal GE, Must A. Relative weight and race influence average age at menarche: results from two nationally representative surveys of US girls studied 25 years apart. Pediatrics. 2003;111(4):844–50. 12671122 10.1542/peds.111.4.844

[50] Freedman DS, Khan LK, Serdula MK, Dietz WH, Srinivasan SR, Berenson GS. Relation of age at menarche to race, time period, and anthropometric dimensions: the Bogalusa Heart Study. Pediatrics. 2002;110(4):e43–e. 12359816 10.1542/peds.110.4.e43

[51] Cortina MA, Sodha A, Fazel M, Ramchandani PG. Prevalence of child mental health problems in sub-Saharan Africa: a systematic review. Archives of pediatrics & adolescent medicine. 2012;166(3):276–81. 22393184 10.1001/archpediatrics.2011.592

[52] Kashala E, Elgen I, Sommerfelt K, Tylleskar T. Teacher ratings of mental health among school children in Kinshasa, Democratic Republic of Congo. European child & adolescent psychiatry. 2005;14(4):208–15. 15981132 10.1007/s00787-005-0446-y

[53] Fazel M, Stein A. Mental health of refugee children: comparative study. Bmj. 2003;327(7407):134 10.1136/bmj.327.7407.134 12869455 PMC165700

[54] Sommer M, Sahin M. Overcoming the Taboo: Advancing the Global Agenda for Menstrual Hygiene Management for Schoolgirls. American journal of public health. 2013;103(9):1556–9. 10.2105/AJPH.2013.301374 23865645 PMC3780686

[55] Winkler I, Roaf V. Bringing the Dirty Bloody Linen Out of its Closet–Menstrual Hygiene as a Priority for Achieving Gender Equality. Forthcoming (2015) Cardozo Journal of Law and Gender. 2014.

[56] Higgins JPT, Green S. Cochrane Handbook for Systematic Reviews of Interventions Version 5.1.0 [updated March 2011]. The Cochrane Collaboration 2011 Available from: www.cochrane-handbook.org.

