# Supplementary material for: Menstruation and the Cycle of Poverty: A Cluster Quasi-Randomised Control Trial of Sanitary Pad and Puberty Education Provision in Uganda
Source: PLoS One. 2016 Dec 21;11(12):e0166122. doi: 10.1371/journal.pone.0166122 (PMC5176162; doi:10.1371/journal.pone.0166122)
Supplement: S2 Table — (PDF) [file pone.0166122.s002.pdf]

S2 Table. School characteristics at baseline

School (site)	School 1	School 2	School 3	School 4	School 5	School 6	School 7	School 8
School fees (UGX/term)	10,000	*Missing*	15,000	7,000	10,000	P1-5: 5,000<br>P6: 10,000<br>P7: 50,000	10,000	7,000
Maize requirement (kg/term)	4	*Missing*	3	3	4	P1-6: 3<br>P7: 5	3	4
Latrines on site: Yes(Y)/No(N)	Y	Y	Y	Y	Y	Y	Y	Y
Latrines with doors: Y/N	Y	Y	Y	Y	Y	Y	Y	Y
Female and male latrines separate: Y/N	Y	Y	Y	Y	Y	N (latrines in same building)	Y	Y
Is there access to clean water on the premises? Y/N	N	N	N	Y	N	N	N	N
How far is the nearest water (approximate estimate) (km)	3	3	1.5	Bore at school	1	2	0.5	2
Are there basins/facilities for washing pads? [1]	None	None	None	None	None (except P7 boarders)	None (except P7 boarders)	None	None
Where do girls go to wash their menstrual absorbent?[1]	Home	Home	Home	Home	Home	Home	Home	Home
Number of girls in the school (all levels) at time of site selection	381	304	170	315	285	304	300	273
School female:male pupil ratio (all levels) at time of site selection	0.8	1.08	1.3	0.88	0.98	1.05	1.16	0.95

[1] This was reported by teachers, but the finding was corroborated in baseline and follow-up surveys, as well as forthcoming qualitative work
