# Supplementary material for: Menstruation and the Cycle of Poverty: A Cluster Quasi-Randomised Control Trial of Sanitary Pad and Puberty Education Provision in Uganda
Source: PLoS One. 2016 Dec 21;11(12):e0166122. doi: 10.1371/journal.pone.0166122 (PMC5176162; doi:10.1371/journal.pone.0166122)
Supplement: S1 File — Trial protocol registration (Pan African Trials Registry PACTR201503001044408) (PDF) [file pone.0166122.s003.pdf]

Pan African Clinical Trials Registry

South African Medical Research Council, South African Cochrane Centre
PO Box 19070, Tygerberg, 7505, South Africa
Telephone: +27 21 938 0506 / +27 21 938 0834 Fax: +27 21 938 0836
Email: pactradmin@mrc.ac.za Website: www.pactr.org

Trial no.:	PACTR201503001044408	Date registered:	2015/02/20
Trial Status:	Retrospective registration - this trial was registered after enrolment of the first participant		

TRIAL DESCRIPTION

Public title	Menstruation and the cycle of poverty
Official scientific title	Menstruation and the cycle of poverty
Brief summary describing the background and objectives of the trial	The proposed research will examine the linkages between menarche, sanitary provisions, reproductive health and girls' educational outcomes in Uganda. Through a randomized trial and qualitative research, our aim now is to provide robust evidence on the effects of sanitary care for girls' educational attainment. The intervention will take place in Kamuli District, located in the eastern-central region of Uganda, where our partner Plan International operates several health and education programmes. The specific objectives of the research are to determine: 1.) whether some combination of puberty education and sanitary pads will improve female educational achievement as measured by attendance, performance, and retention; 2.) whether puberty education and/or sanitary pads will improve girls¿ confidence and concentration, subjective well-being, and participation in community activities; 3) whether the purchase preferences, customs, and attitudes of households related to sanitary products act as a barrier to girls¿ education; and 4) whether sanitary pad disposal causes untoward environmental implications
Type of trial	RCT
Acronym (If the trial has an acronym then please provide)	
Disease(s) or condition(s) being studied	Menstruation
Purpose of the trial	Education/Counselling/Training
Anticipated trial start date	2011-09-01
Actual trial start date	2011-09-01
Anticipated date of last follow up	2014-11-30
Actual date of last follow up	2014-11-30
Anticipated target sample size (number of participants)	560
Actual target sample size (number of participants)	1124
Recruitment status	Closed to recruitment: follow up complete
Publication URL	

Secondary Ids	Issuing authority/Trial register	Links to Secondary ID

STUDY DESIGN

Intervention assignment	Allocation to intervention	If randomised, describe how the allocation sequence was generated	Describe how the allocation sequence/code was concealed from the person allocating the participants to the intervention arms	Masking	If masking / blinding was used
Factorial: participants randomly allocated to either no,one,some or all	Randomised		Computer software randomisation	Open-label (masking	

interventions simultaneously				not used)	

INTERVENTIONS						
Intervention type	Intervention name	Dose	Duration	Intervention description	Group size	Nature of control
Experimental group	Afripads	Pack given	18m	Sanitary pads with soap	140	
Experimental group	Education only	single session	2h	standardised education about puberty and menstruation	140	
Experimental group	Education plus Afripads	Pack given	18m	Single session 2h plus sanitary pads with soap	140	

ELIGIBILITY CRITERIA				
List inclusion criteria	List exclusion criteria	Min age	Max age	Gender
Enrolled in school Schools separated to avoid contamination Willing to participate and work with NGO	Schools separated to avoid contamination	12 Years	15 Years	Female

ETHICS APPROVAL

Has the study received appropriate ethics committee approval	Date the study will be submitted for approval	Date of approval	Name of the ethics committee
Yes		2012/01/16	SOCIAL SCIENCES & HUMANITIES INTER-DIVISIONAL RESEARCH ETHICS COMMITTEE

Ethics Committee Address			
Street address	City	Postal code	Country
Hayes House, 75 George Street	Oxford	OX12BQ	United Kingdom

Has the study received appropriate ethics committee approval	Date the study will be submitted for approval	Date of approval	Name of the ethics committee
Yes		2014/01/17	The AIDS Support Organisation (TASO)

Ethics Committee Address			
Street address	City	Postal code	Country
P.O. Box 10443	Kampala		Uganda

OUTCOMES		
Type of outcome	Outcome	Timepoint(s) at which outcome measured
Primary Outcome	School attendance	Baseline Midpoint Follow up 18m
Secondary Outcome	Subjective well being	Baseline Midpoint Follow up 18m
Secondary Outcome	Indicators of agency, confidence, empowerment and a range of cultural beliefs	Baseline Midpoint Follow up 18m

RECRUITMENT CENTRES				
Name of recruitment centre	Street address	City	Postal code	Country
Kumuli Uganda	Plan International	Kumuli	UG-205	Uganda

FUNDING SOURCES				
Name of source	Street address	City	Postal code	Country
Economic and Social Research Council UK	Polaris House	Swindon	sn2 1uj	United Kingdom

SPONSORS

Sponsor level	Name	Street address	City	Postal code	Country	Nature of sponsor
Primary Sponsor	University of Oxford	Wellington Square	Oxford	ox1 2er	United Kingdom	University

COLLABORATORS

Name	Street address	City	Postal code	Country
Dr Catherine Dolan	Said Business School	Oxford	ox1 1hp	United Kingdom
Prof LInda Scott	Said Business School	Oxford	ox1 1hp	United Kingdom
Prof Sue Dopson	Said Business School	Oxford	ox1 1hp	United Kingdom
Plan International Uganda	Kumuli	Kumuli	UG-205	Uganda

CONTACT PEOPLE

Role	Name	Email	Phone	Fax
**Principal Investigator**	Prof Paul Montgomery	paul.montgomery@ spi.ox.ac.uk	44 1865 270325	44 1865 270324

Street address	City	Postal code	Country	Position / Affiliation
Centre for Evidence Based Intervention, University of Oxford	Oxford	OX1 2ER	United Kingdom	Professor of Psycho-Social Intervention

Role	Name	Email	Phone	Fax
**Public Enquiries**	Prof Linda Scott	linda.scott@sbs.ox.ac.uk	44 1865 288800	

Street address	City	Postal code	Country	Position / Affiliation
Said Business School	Oxford	ox1 1hp	United Kingdom	DP World Chair in Marketing

Role	Name	Email	Phone	Fax
**Scientific Enquiries**	Prof Paul Montgomery	paul.montgomery@ spi.ox.ac.uk	44 1865 270325	44 1865 270324

Street address	City	Postal code	Country	Position / Affiliation
Centre for Evidence Based Intervention, University of Oxford	Oxford	OX1 2ER	United Kingdom	Professor of Psycho-Social Intervention

Changes to trial information

Date	Reason	Old Value	Update Value
2016-10-20 16:19:00.0	A larger sample was included to improve power.		1124
2016-10-20 16:20:00.0	Inclusion criteria changed to include girls pre- and post-menarche	Menstruating girls Enrolled in school Schools separated to avoid contamination Willing to participate and work with NGO	Enrolled in school Schools separated to avoid contamination Willing to participate and work with NGO
